# Machine Learning in Homogeneous Catalysis: Basic Concepts and Best Practices

**DOI:** 10.1021/acscatal.5c06439

**Published:** 2025-12-19

**Authors:** David Dalmau, Susana García-Abellán, Juan V. Alegre-Requena

**Affiliations:** Departamento de Química Inorgánica, Instituto de Síntesis Química y Catálisis Homogénea (ISQCH), 16379 CSIC-Universidad de Zaragoza, C/Pedro Cerbuna 12, 50009 Zaragoza, Spain

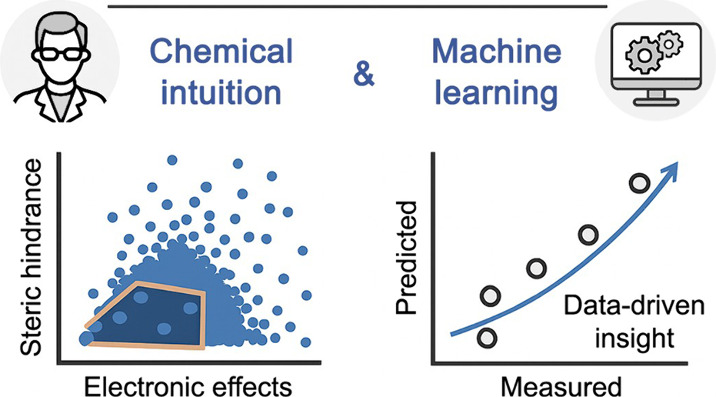

## Introduction

Recent advances in machine learning (ML) offer new opportunities for homogeneous catalysis, from accelerating discovery to optimizing performance and enabling sustainable design.^1,2^ However, the development of new catalysts in this domain remains predominantly empirical, with a limited integration of data-driven methodologies. As a result, progress is still largely guided by intuition and trial-and-error approaches that differ little from those used decades ago.

One of the main barriers preventing the widespread adoption of ML is educational, as most chemists lack formal training in data science and ML techniques can seem out of their reach.^3,4^ In addition, the supporting ecosystem, including curated datasets, user-friendly tools, and clear benchmarking practices, is still underdeveloped or scattered across domains.^5^ This situation limits the use of ML to a small number of specialized groups, while its integration into routine experimental workflows is still uncommon.^6^

This Viewpoint offers a set of guidelines and considerations for implementing ML tools in homogeneous catalysis in a way that is both rigorous and accessible. The manuscript is particularly suited for experimental and computational chemists with little to no experience in ML who intend to apply this technology in their research. The protocols discussed are particularly useful for projects involving dozens to hundreds of experiments or calculations, a common scenario in the field.

Herein, we outline how to represent molecules in a way that is computer readable, structure datasets, and apply ML models that balance predictive power with interpretability. Through selected case studies, we highlight applications commonly encountered in homogeneous catalysis where chemists can benefit from data-driven strategies, particularly in substrate and catalyst sampling or discovery.

Our aim is to help chemists engage more critically and effectively with ML, not just as tool users but as informed practitioners aware of its possibilities and limitations. Ultimately, we argue that the thoughtful integration of ML can transform catalysis, not by replacing chemical intuition but by enhancing it with data-driven insight. While this work focuses on homogeneous catalysis, it is simply an illustration of how coupling ML with chemistry can drive significant breakthroughs across the broader chemistry and materials domains.

## FIRST STEP: DIGITALIZATION OF MOLECULES

One of the first steps in enabling ML predictions is to represent molecules in a way that is computer readable. When chemists examine a molecule, they intuitively recognize functional groups, electronic effects, and steric environments (Figure 1). For example, a nitro group (−NO_2_) is electron-withdrawing, a *tert*-butyl group (−*^t^* Bu) introduces steric hindrance, and a metal center modulates reactivity through its ligand environment. These concepts, while intuitive to humans, are meaningless to an algorithm unless translated into numerical values.

**Figure 1:**
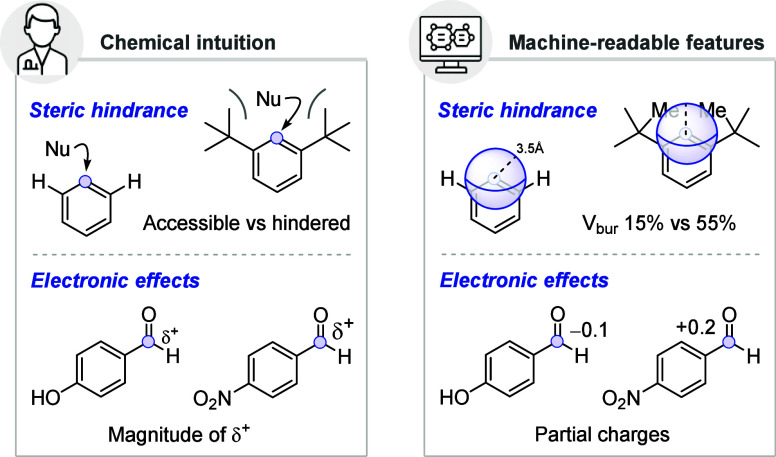
Examples of transforming concepts from chemical intuition (left) into machine-readable descriptors (right).

This translation process, known as featurization, represents the first critical step in applying ML to catalysis,^7–10^ as it is the conceptual bridge between chemical intuition and data-driven modeling. For instance, if chemists want a ML model to quantify the steric hindrance of a target reactive site, they can use numerical descriptors such as buried volume (*V*_bur_).^11^ This descriptor was originally developed to describe the steric demand of ligands around metal centers in organometallic complexes,^12^ but it can be adapted to evaluate steric environments around any atom (Figure 1, top right). Similarly, for electronic effects, chemists need to calculate features such as electrostatic potential (ESP) or atomic charges. These values help capture whether a ring is electron-rich (i.e., with a −NMe_2_ group) or electron-poor (i.e., with a −NO_2_ group), transforming chemical intuition into machine-readable data that a model can interpret (Figure 1, bottom right).

Descriptors can capture the properties of an entire molecule (molecular descriptors) or specific atoms within the molecule (atomic descriptors). In ML models for homogeneous catalysis, molecular descriptors are often complemented by atomic descriptors, as catalytic activity and selectivity frequently depend on the specific local environment around reactive sites.^13^ Importantly, chemical intuition plays a central role in selecting which descriptors to generate, since the performance of ML models are strongly influenced by the relevance of the input features.^14^ For example, a chemist experienced in Michael additions understands that the reaction outcome largely depends on the electrophilicity and steric accessibility of the carbon atom undergoing nucleophilic attack. Failing to include descriptors for this atom or introducing irrelevant features will likely reduce the quality of the resulting model, even if performance metrics appear high.

To capture these local effects in an ML framework, atomic descriptors for that carbon should contain electronic properties (i.e., partial charges, Fukui indices,^15^ etc.) and steric parameters such as *V*_bur_ or solvent-accessible surface area (SASA).^16^ Similarly, in metal-catalyzed reactions, the metal center often dominates reactivity, and descriptors that capture the electronic and steric properties of that atom are essential for building accurate predictive models. In such cases, the properties of the coordinating atoms of the ligands are often used instead of those of the metal itself. For example, the phosphorus atom in phosphine ligands or the nitrogen atoms in pyridines may be used to obtain representative descriptors. This approach enables simpler featurization protocols while still capturing the key characteristics of the catalytic center.

For nonspecialists, the simplest entry point into descriptor generation might be the use of online descriptor databases with quantum-mechanical (QM) features for large collections of ligands and catalysts. These tools offer researchers easy access to rich electronic and steric information without requiring custom calculations or coding skills. However, such databases typically cover only a limited range of structures and may be of limited use when the goal is to design new ligands. In practice, especially in catalysis, descriptors are often generated on-demand by using QM methods. Density functional theory (DFT) and semiempirical approaches such as GFN2-xTB^17^ are increasingly popular for calculating descriptors relevant to catalytic performance, including atomic charges, HOMO–LUMO energies (where HOMO denotes highest occupied molecular orbitals and LUMO denotes lowest unoccupied molecular orbitals), and Fukui indices, while steric parameters can be derived from the optimized QM structures.

This QM strategy is particularly valuable when experimental data are scarce, as is often the case in catalysis, where only a few dozen experiments may be available. However, when working with large-scale datasets, such as those generated in digital high-throughput screenings, QM calculations become computationally prohibitive. In these scenarios, alternative strategies such as molecular fingerprints from libraries like RDKit^18^ or feature-learning methods based on graph neural networks (GNNs)^19^ allow the rapid featurization of millions of molecules within reasonable computational times. These scalable methods are powerful when data are abundant, but they come with tradeoffs: they often require greater programming expertise and generally lack the interpretability of physics-based descriptors.^20^ Consequently, while fingerprints and GNNs are highly effective for large, high-volume datasets, they are rarely the first choice for the smaller, carefully curated datasets that are typical in catalysis research.

More information, implementation details, guidelines, and considerations about descriptor generation are shown in Tables 1 and 2.

**Table 1:**
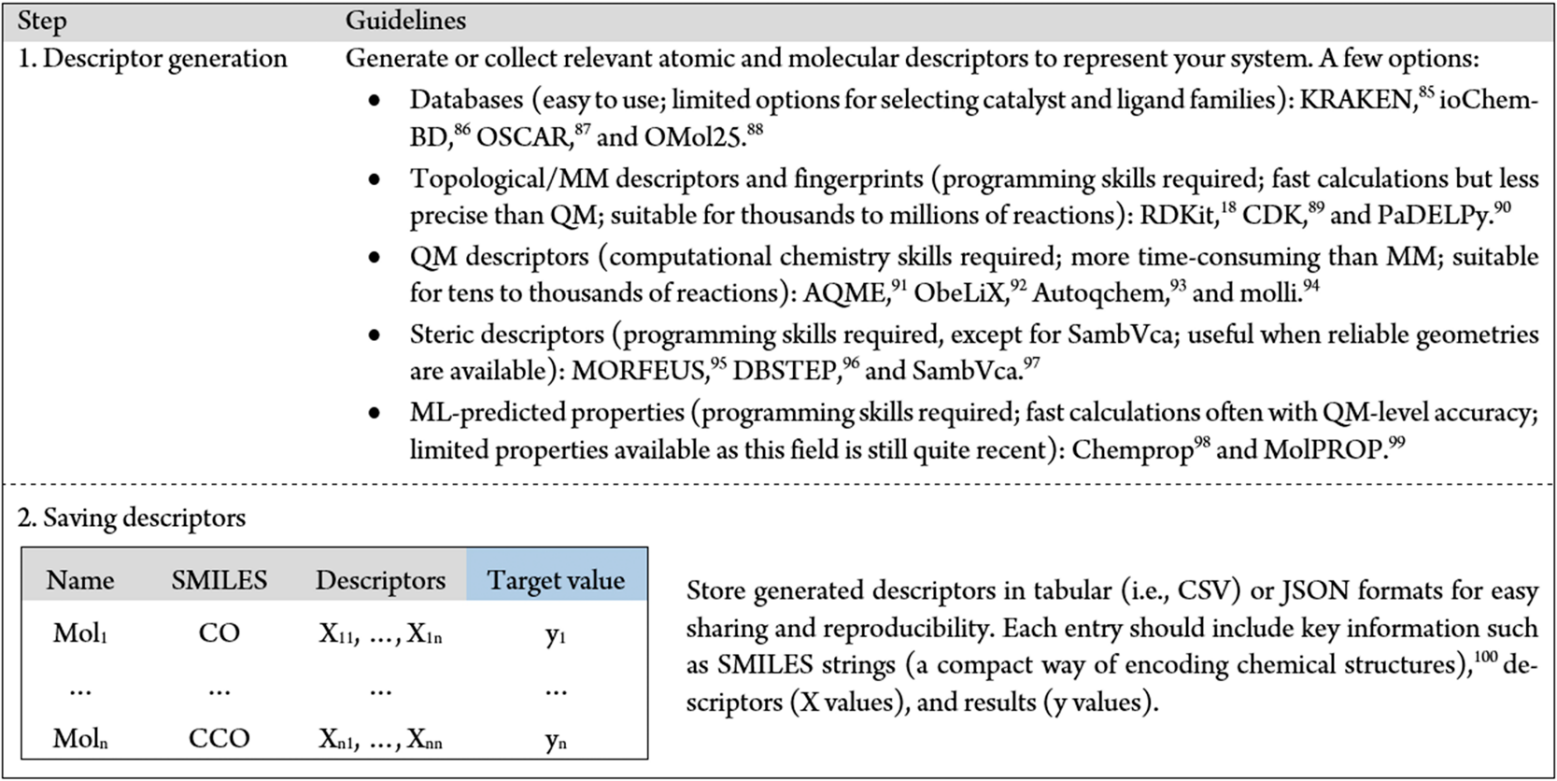
Guidelines for Generating Descriptors^85–100^

**Table 2:**
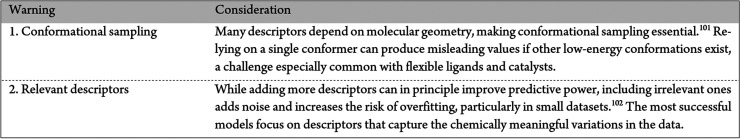
Warnings and Considerations for Descriptor Generation^101,102^

## SAMPLING SUBSTRATES AND CATALYSTS WITH DATA-DRIVEN CLUSTERING

One of the most common pitfalls of human intuition arises in selecting substrate scopes,^21^ a standard practice used to evaluate the generality of a catalytic method. In most articles, researchers tend to follow well-established patterns of chemical intuition to study how applicable the method is. They often explore electronic effects with a one-variable-at-a-time strategy, such as substituting aromatics with a *para* electron-withdrawing group (−NO_2_, −CF_3_, −CN), hydrogen, or electron-donating group (−OMe, −NMe_2_). Similarly, steric effects are usually probed by replacing substituents such as −H, −Me, −^*i*^Pr, and −^*t*^Bu at a single position close to the reactive center. While this approach provides a general sense of how structural modifications influence catalytic activity, it represents an inefficient way of exploring chemical space and yields results that cover only a small fraction of the available diversity.

As an illustrative example, we downloaded and curated a set of 25 048 commercially available bromoaryl (Ar–Br) substrates from Enamine, the chemical supplier, specifically from a collection designed for Pd-catalyzed cross-couplings.^22^ We then mapped the chemical space^23^ of substrates available from this vendor using two key descriptors: an electronic property (the partial charge on the leaving Br atom) and a steric parameter (the *V*_bur_ of Br).^24^ The chemical space is represented with blue points in the graphs of Figure 2. For comparison, we examined two reported metal-catalyzed cross-couplings of aromatic halides, case A^25^ and case B,^26^ which involved 19 and 12 substrates, respectively.

**Figure 2:**
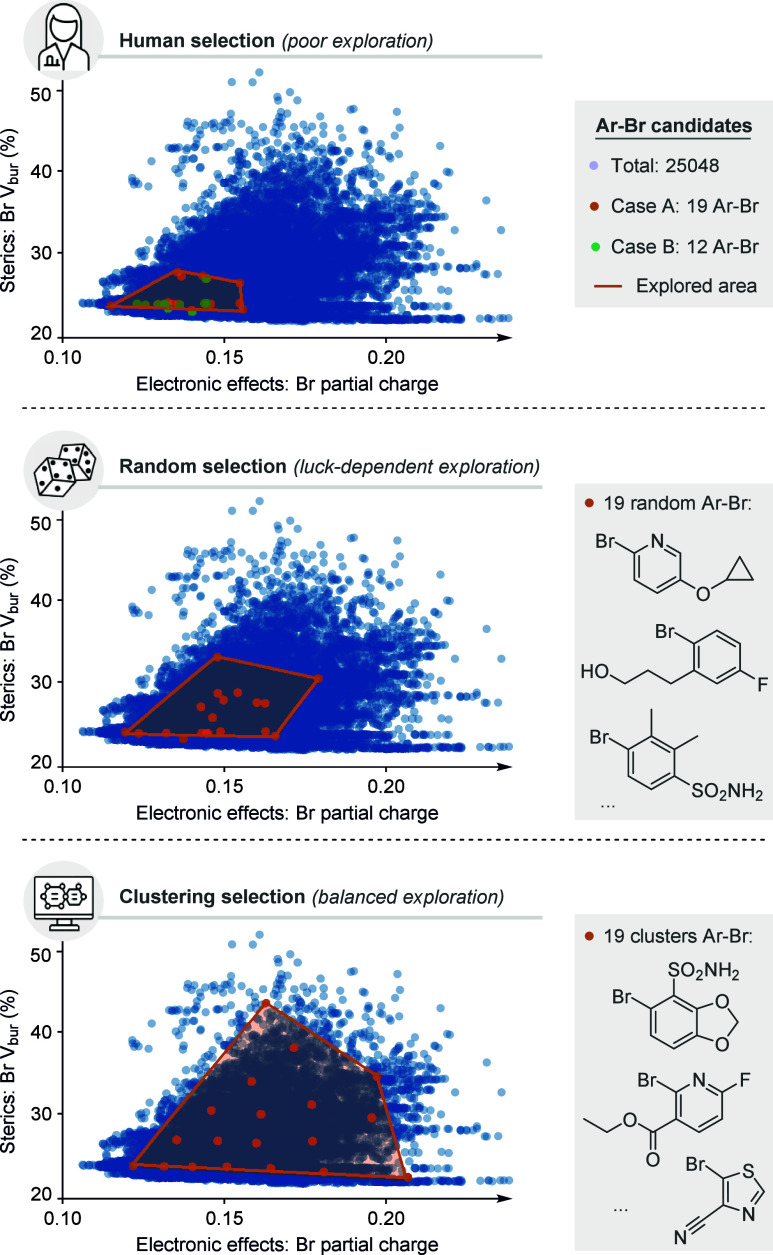
Chemical space exploration through human-guided (top), random (middle), and data-driven (bottom) sampling.

In both cases, the chosen molecules occupy only a small region of the vast and diverse chemical space (see the orange area in the top portion of Figure 2), underscoring the limited scope and generality of the catalytic methods. Although these two cases serve purely as illustrative examples, similar trends likely extend across much of the cross-coupling literature, suggesting that relying solely on reported examples may provide an incomplete view of catalytic generality and limitations.

Based on this result, one might argue that, once such a dataset of substrates is created, even random selection strategies could often explore a more diverse region of chemical space. In the example shown in Figure 2, middle, we picked substrates at random^27^ and observed a considerable increase in the coverage of the explored region, although it still represented only a limited portion of the total space. A key drawback of this approach, however, is that random selections are inherently dependent on the chosen random seed, and whether the sampled substrates cover a wide chemical space is essentially a matter of luck.

To overcome the inefficiencies of traditional substrate selection, unsupervised ML methods such as clustering are gaining traction in the chemistry community,^28^ with notable applications from the Sigman,^29^ Doyle,^30^ and Glorius groups,^21^ among others. Clustering algorithms require only descriptors, without any pre-existing activity data, to partition the chemical space into groups of compounds with similar properties. The resulting clusters reveal natural groupings without prior bias, and representative candidates can then be selected for targeted experimental validation.

An illustrative example using 38 Ar–Br structures and two properties (*V*_bur_ and the partial charge of Br) is shown in Figure 3. In this example, the molecules are separated into three clusters. Cluster A consists of phenyl rings with electron-donating groups in the *para* position, relative to the Br atom and no proximal bulky substituents, corresponding to a low partial charge and low *V*_bur_ at the Br atom. Cluster B contains rings where the Br atom is flanked by bulky and electron-donating groups, appearing in the upper left region of the plot. Cluster C includes aromatics with *para* electron-withdrawing groups relative to the Br atom and no nearby steric repulsions located in the lower right region of the graph.

**Figure 3:**
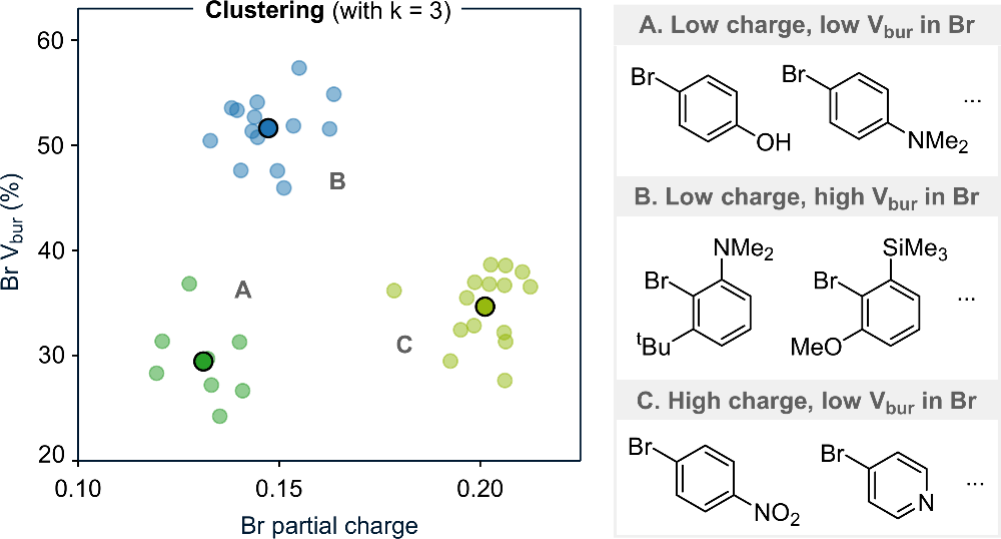
Example of a clustering result with three clusters (left) and the molecular properties associated with each cluster (right).

Once the clusters are defined, the point closest to the centroid of each cluster can be selected as a representative molecule for sampling (Figure 3, points with black borders). Using the larger-scale example from Figure 2, we generated 19 clusters using a *k*-means clustering algorithm^31^ and then selected the corresponding centroid molecules for comparison with the original 19 substrates from case A. Unlike intuition-driven selection, which is often guided by prior experience and limited to low-dimensional changes, clustering approaches objectively capture high-dimensional descriptor relationships and enable more efficient chemical exploration (Figure 2, bottom). By selecting a small set of substrates from distinct clusters, chemists can efficiently probe a more diverse and representative chemical space, ultimately gaining deeper insight into the generality and limitations of their catalytic methods.

In addition to guiding substrate selection, clustering can also be applied to explore catalyst diversity, a common strategy in homogeneous catalysis for optimizing the reactivity and selectivity. Different groups have adopted this data-driven methodology, with notable examples from the Pérez-Ramírez,^32^ Jorner,^33^ Ackermann,^34^ and Denmark^35^ groups, among others.

Clustering has also been employed to discover catalysts by incorporating experimental results into generated clusters. For example, Schoenebeck and co-workers constructed a chemical space of ligands for the synthesis of palladium­(I) dimers, which are challenging to stabilize.^36^ Using an *k*-means algorithm, they divided ligand space into groups with related properties and subsequently incorporated stability data from five ligands. This analysis revealed that certain clusters were enriched in active ligands, whereas others contained inactive ones. This relatively simple strategy enabled the digital exploration of new phosphine ligands within the chemical space, and the prioritization of candidates from clusters containing stable representatives. Experimental validation ultimately led to the discovery of eight previously unexplored Pd dimers under conditions of very limited available data.

As a final remark for this section, it is important to note that more than three descriptors are typically used to featurize molecules and, consequently, to define chemical spaces. In such cases, the principal components obtained through principal component analysis (PCA)^37^ can be employed to represent the chemical space in two- or three-dimensional plots. Plotting the sampling selection in these graphs helps to verify that there is sufficient coverage of the chemical space. When using this approach, researchers should ensure that the selected principal components together capture a substantial portion of the dataset’s variance (typically 60%–70% or more).^38^ Alternatively, more advanced nonlinear dimensionality reduction techniques, such as UMAP^39^ and t-SNE,^40^ are often preferred over PCA for visualizing chemical space and guiding sample selection.^41^

Guidelines and considerations relevant to this section are summarized in Tables 3 and 4 and should be carefully reviewed before attempting any clustering-based sampling.

**Table 3:**
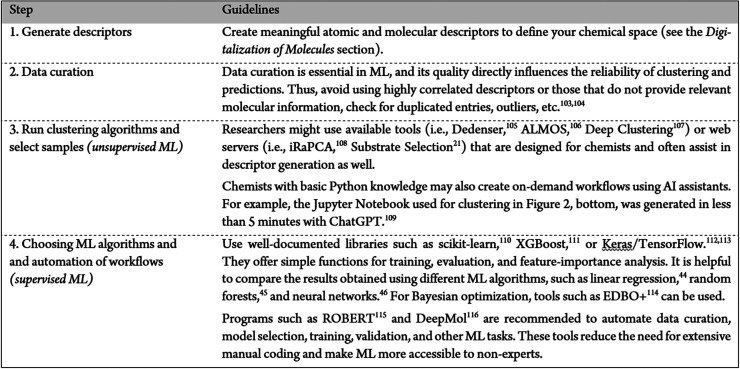
Guidelines for Sampling with Clustering and for Supervised ML^21,44–46,103–116^

**Table 4:**
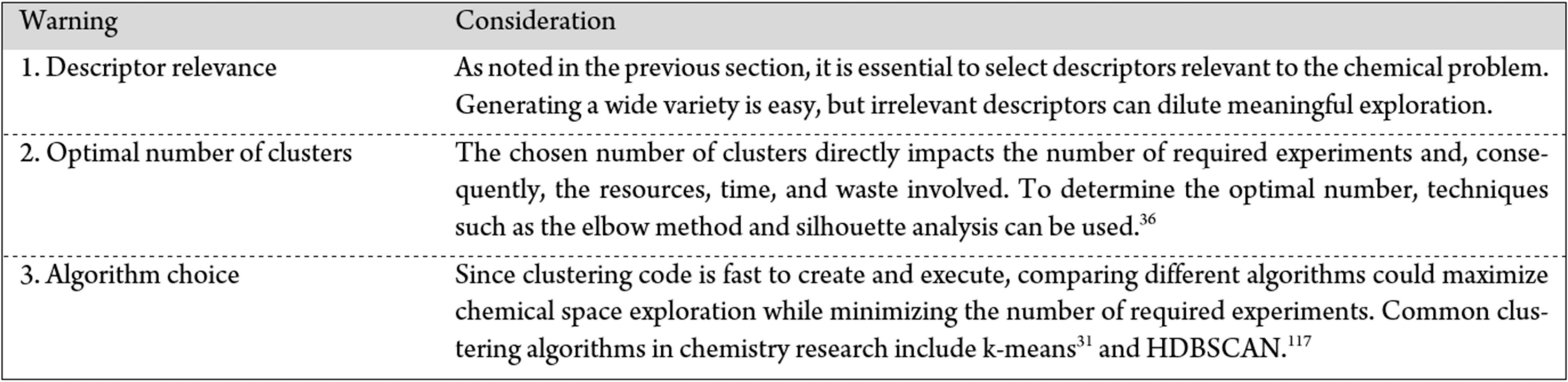
Warnings and Considerations for Clustering^31,36,117^

## CATALYST DISCOVERY WITH SUPERVISED ML

Supervised learning refers to the development of predictive models from datasets that contain descriptors as the *X* matrix (i.e., electronic properties, steric features, etc.) and one or more outcomes as the *y* matrix (i.e., reactivity, selectivity).^42,43^ Broadly, the *X* matrix is processed by ML algorithms such as linear regression,^44^ random forests,^45^ and neural networks,^46^ which learn from the descriptor data to predict the *y*-values. In catalysis, this learning of patterns across datasets makes it possible to map complex relationships between reaction parameters and performance metrics, enabling smarter experimental design and accelerating the discovery of optimal catalysts and conditions.^3,47^

The target catalytic properties (*y*-values) may be obtained from either experimental measurements or computational calculations. Experimentally, catalytic activity is often quantified through yields, and turnover numbers and frequencies,^48,49^ while selectivity is typically expressed using metrics such as enantiomeric excess and diastereoselectivity or regioselectivity ratio.^50,51^ Computationally, the most common approach involves DFT calculations, which provide energy barriers for competing pathways. These data allow direct computation of parameters such as Δ*G* ^⧧^ or Δ*E* ^⧧^ for reaction rates, and ΔΔ*G* ^⧧^ or ΔΔ*E* ^⧧^ for selectivity.^52,53^

Supervised learning problems in catalysis can generally be divided into regression and classification tasks. In regression, the objective is to predict numerical values within a continuous range, such as yields, rate constants, or enantioselectivity. In contrast, classification problems aim to predict discrete outcomes, such as whether a catalyst is active or inactive, or to categorize performance levels (i.e., high vs low selectivity).^54^ Notably, even small datasets containing as few as 18 reactions can deliver reliable predictions, provided that the chosen descriptors effectively capture the key chemical information.^55^

In both regression and classification problems, two main data-driven strategies are typically used for catalyst discovery: rational catalyst design enabled by explainable ML, and ML-based candidate prediction (Figure 4). These strategies are often integrated into iterative active learning cycles. In the first case, Strategy A employs a predictive model combined with SHapley Additive exPlanations (SHAP) feature analysis,^56^ enabling chemists to gain mechanistic insights and prioritize the most informative candidates for testing through rational design. In the example from the figure, the SHAP analysis reveals that reduced steric hindrance and lower charge at the Pd centers (Pd charge and Pd *V*_bur_) correlate with higher yields. This insight can help chemists to propose improved catalysts by incorporating groups that minimize these properties. A landmark example by Sigman and co-workers used multivariate linear regression to correlate steric and electronic descriptors of BINOL-derived phosphoric acids with experimental enantioselectivities, enabling accurate out-of-sample predictions (beyond the training set) and guiding ligand selection across diverse substrates.^57^

**Figure 4:**
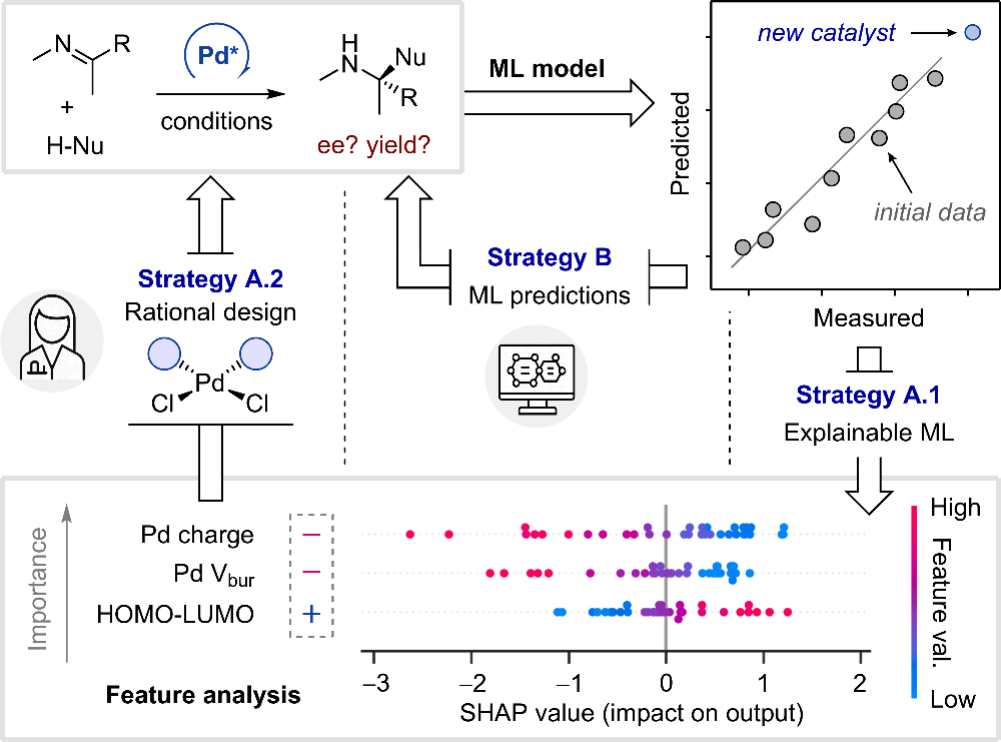
Comparison of different data-driven catalyst discovery strategies: rational design versus ML-based suggestions.

Alternatively, Strategy B focuses on discovering catalysts with minimal experimentation by relying solely on ML-guided suggestions. This strategy often employs Bayesian optimization (BO)^58^ to prioritize experiments, balancing options expected to work best (exploitation) with less certain choices that can provide new insights (exploration). In catalysis, this approach maximizes information gained per experiment while minimizing redundant trials. This discovery strategy is particularly popular for optimizing reaction conditions using descriptor matrices that include variables such as the temperature, catalyst loading, and solvent. As an example, Doyle and co-workers demonstrated that BO identified high-yielding reaction conditions for a Pd-catalyzed arylation reaction faster than human chemists, highlighting its efficiency in navigating complex reaction landscapes.^59^

While this section highlights the advantages of using supervised ML and encourages its adoption, it is important for readers to understand that great care must be taken when developing and applying this technology. A double-edged situation is that many tools now allow chemists to build ML workflows and obtain predictions within minutes, regardless of their programming or data science expertise. Although this accessibility is crucial for the broader adoption of ML, the combination of limited expertise and the ease of model generation can be problematic, as it becomes relatively easy to overestimate the quality or predictive power of our tools. For example, a researcher might train a model with a correlation coefficient of *R* ^2^ = 0.95. These results could misleadingly suggest strong performance, when, in fact, the model is overfitted but went undetected because proper overfitting tests were skipped.

To avoid these pitfalls, researchers should evaluate models using multiple metrics (i.e., *R* ^2^, RMSE, MAE, accuracy, F1 score, and Matthews correlation coefficient) rather than relying on a single measure of performance. In addition, held-out test sets and *k*-fold cross-validation (preferably 5-fold CV) should be used to assess consistency and detect overfitting. Leave-one-out CV (LOOCV) can be applied to very small datasets but should be used with caution, as it is more brittle for detecting overfitting, compared to 5-fold CV.^60^ One example of an evaluation technique that combines these analyses with additional statistical tests (i.e., y-shuffle, y-mean, extrapolation) is the ROBERT score.^61^ This metric evaluates models on a 10-point scale, considering three key aspects: predictive ability and overfitting, prediction uncertainty, and detection of spurious predictions.

Another essential consideration is the predictive scope of ML models, as they are often applied to predict outcomes for molecules beyond the range of their training data, where reliability drops sharply.^62^ For instance, a researcher might develop an excellent predictor for the yields of substitution reactions in pyridines, but the same model may fail for pyrazines, even if intuition suggests otherwise. In general, predictors should be treated with caution when applied to extrapolated regions outside their training sets, and experimental validation is strongly recommended in these cases. Examples of extrapolation include predicting yields higher than those observed in the training set (Figure 5A) and predicting outcomes for molecules substantially different from those used for training (Figure 5B). When ML predictors fail to extrapolate reliably, focusing on interpolation within the dataset used to train the model can still yield useful insights. For example, such models can be used to discover reactivity trends and identify molecular features that are relevant to a particular set of catalytic results.

**Figure 5:**
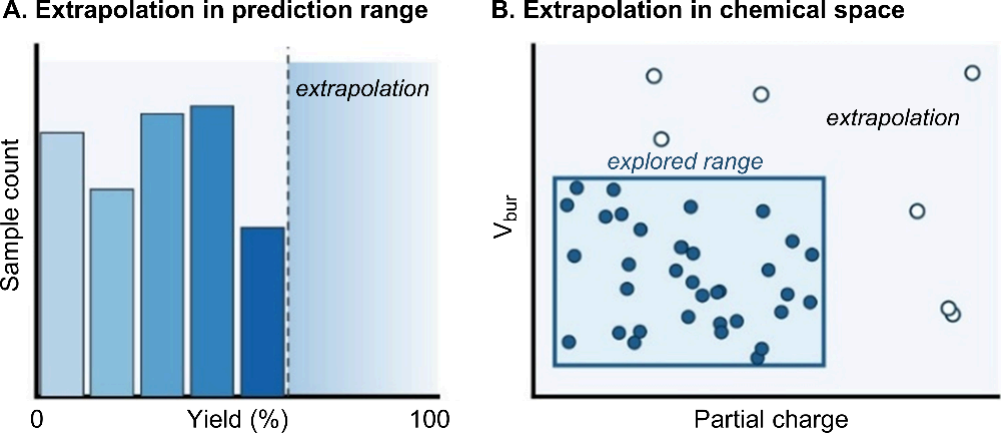
Different types of extrapolation in supervised ML with respect to (A) the prediction range and (B) the chemical space.

Lastly, it is important to recognize that the model accuracy depends largely on the quality of the training data. Studies on yield prediction consistently show a performance gap between models trained on high-throughput experimentation (HTE) datasets and those trained on electronic laboratory notebook (ELN) records.^63^ HTE datasets typically follow strict, standardized protocols for reaction setup, analysis, and data logging, resulting in clean, balanced datasets with minimal missing information. By contrast, ELN-derived data are often accumulated over years by different researchers and tend to suffer from heterogeneous formats, incomplete metadata, and inconsistent experimental practices. For these reasons, another key consideration is that datasets compiled from different manuscripts and patents often contain noisy yield values, which can severely undermine the accuracy of ML models.

In this context, chemists should, whenever possible, report crude yields (before purification) for comparability and maintain consistent conditions such as temperature, solvent, and sampling time when building or merging datasets. Reactions should also be performed in at least duplicate to ensure reproducibility and avoid spurious results. While selectivity might be less sensitive to some reaction parameters, caution is still recommended (i.e., diastereomeric ratios may change after column chromatography purification). Moreover, yield, conversion, and sometimes selectivity are strongly time-dependent (Figure 6A). It is therefore essential to select meaningful reaction times or compare results across multiple time points.

**Figure 6:**
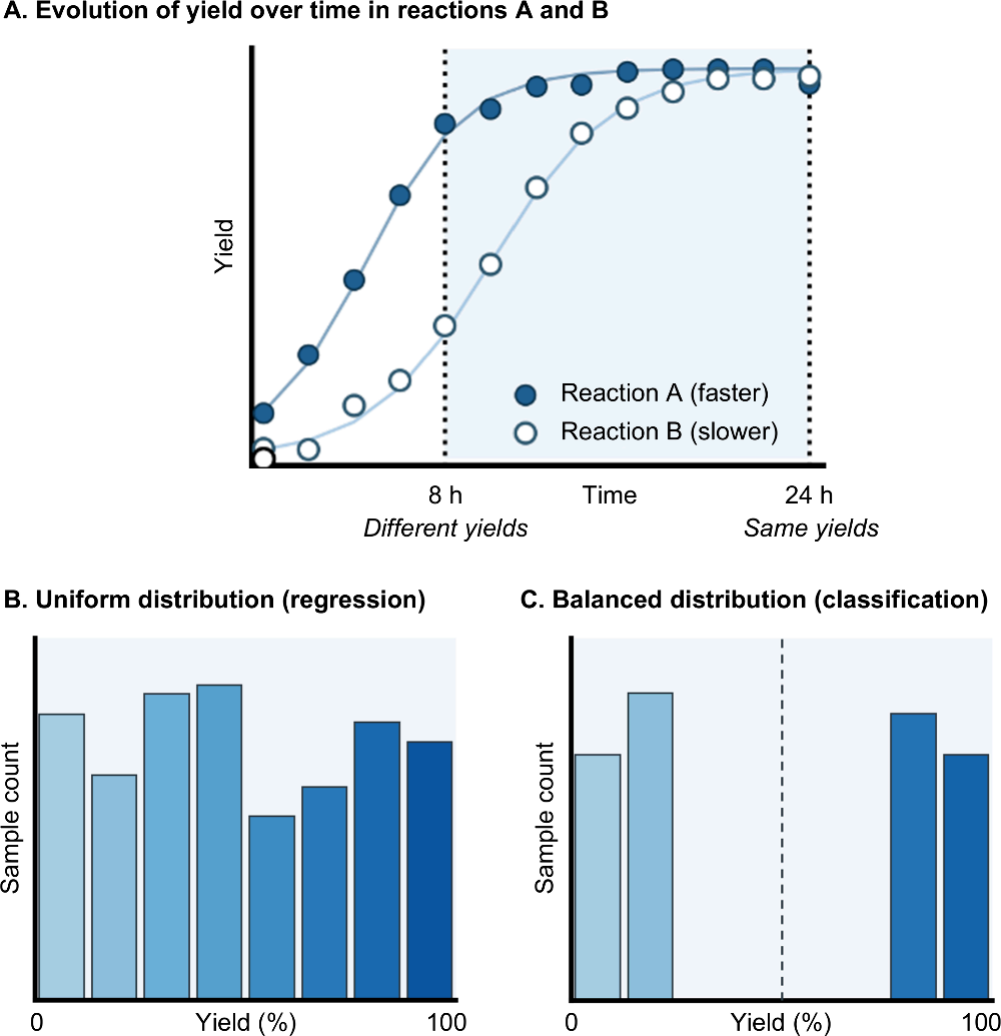
(A) Yield evolution over time in two reactions with different kinetics. Optimal distribution of target values for (B) regression and (C) classification.

For optimal performance, datasets should be balanced to minimize bias and improve model robustness. In regression, a uniform distribution of target values is desired (Figure 6B) so the model learns across low, medium, and high regions. In classification, ensure similar numbers of samples per class (Figure 6C) to avoid bias and loss of generalization. When data are highly skewed or concentrated around a single value, targeted data acquisition may be necessary to restore balance before modeling.^64^ In a similar context, poor performance results or “negative data” (i.e., low yields, enantioselectivities, etc.) are encouraged to be reported and included in models to broaden the scope of the algorithms and make them more general and robust.^5,65^

Further guidelines and considerations are summarized in Tables 3 and 5.

**Table 5:**
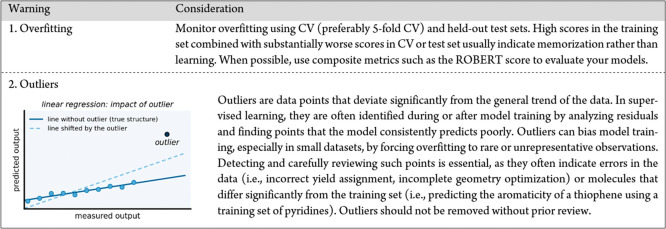
Warnings and Considerations for Supervised ML

## ADVANCED ML APPLICATIONS IN CATALYSIS, FUTURE DIRECTIONS, AND FURTHER READING

Despite the significant potential of ML to accelerate catalytic studies, its adoption within the chemistry community remains limited and, at times, misapplied, particularly when predictive models are used without an adequate understanding of their limitations. A common reason for these limitations is that implementing ML often requires time and effort to gain expertise in fields outside of chemistry (i.e., data science, programming). Closing this gap will require both education and rigor, including initiatives to embed hands-on digital chemistry modules into university curricula, offer workshops on ML applications, develop more user-friendly software, and adopt the Findable, Accessible, Interoperable, and Reusable (FAIR) principles.^66^ In other cases, ML remains underutilized because researchers are skeptical. However, as with other disruptive technologies throughout history, this mistrust will likely fade as further advances emerge.

In the context of ML research, one of the most promising directions is the development of automated robotic platforms, which pave the way for the popularization of self-driving laboratories. Even though this is still a young field, several representative examples have already demonstrated success in catalytic reaction discovery, including work from the groups of Cooper,^67^ Aspuru-Guzik,^68^ and Noël,^69^ among others.

Another emerging technology with a growing influence in ML-driven catalysis is the use of large language models (LLMs). The creation of chatbot assistants holds great promise, as they can help chemists generate code, extract descriptors from the published literature, and guide digital catalyst discovery through natural language conversations. Promising results have already been reported by the teams of Gomes,^70^ White,^71^ Laino,^72^ and Schwaller,^73^ among others.

For descriptor generation, a particularly exciting development is the emergence of machine learning potentials (MLPs), which enable simulations of catalytic systems with near-DFT accuracy at a fraction of computational cost. While they are still at an early stage of adoption in catalysis, MLPs are poised to transform QM calculations and facilitate the routine exploration of complex reaction landscapes. Notable contributions in this area have been made by the groups of Isayev,^74^ Dral,^75^ Duarte,^76^ and Wood and Zitnick,^77^ among others.

An additional promising direction is ML-based inverse design, which aims to generate in silico catalysts by suggesting structural modifications through algorithms. These methods are often combined with filtering strategies to eliminate candidates that are too expensive or synthetically unfeasible. Active groups in this area include those of Balcells,^78^ Jensen,^79^ and Bhowmik,^80^ among others.

As a final remark, it is worth mentioning that the intention of this viewpoint is to introduce readers to some useful concepts and capabilities that ML can offer to those working in homogeneous catalysis. Its format is intentionally short and concise, aiming to encourage the adoption of ML and to popularize this technology within the broader catalysis community. For this reason, further reading is encouraged for those seeking a deeper understanding of ML before applying it in research intended for publication, including reviews and studies from the Illas,^1^ Schuurman,^2^ Fey,^81^ Norrby,^82^ Maseras,^83^ Shimizu,^84^ and Wiest^63^ groups, among others.

## Data Availability

Raw data, instructions, and code used for descriptor generation and clustering are freely available on Zenodo (https://zenodo.org/records/17084325).
